# Silibinin Suppresses TNF-α-Induced MMP-9 Expression in Gastric Cancer Cells through Inhibition of the MAPK Pathway 

**DOI:** 10.3390/molecules14114300

**Published:** 2009-10-26

**Authors:** Sangmin Kim, Min Gew Choi, Hye Sook Lee, Se Kyung Lee, Sung Hoon Kim, Wan Wook Kim, Sung Mo Hur, Jung-Han Kim, Jun-Ho Choe, Seok Jin Nam, Jung-Hyun Yang, Sung Kim, Jeong Eon Lee, Jee Soo Kim

**Affiliations:** Department of Surgery, Samsung Medical Center, Sungkyunkwan University School of Medicine, 50 Ilwon-dong, Kangnam-gu, Seoul, Korea

**Keywords:** silibinin, TNF-α, MMP-9, gastric cancer

## Abstract

Tumor necrosis factor (TNF)-α is one of the pro-inflammatory cytokines highly expressed in *Helicobacter pylori* that inhibits gastric acid secretion. In this study we determined the effect of silibinin on TNF-α-induced MMP-9 expression in gastric cancer cell lines. MMP-9 mRNA and protein expression was dose-dependently increased by TNF-α in SNU216 and SNU668 gastric cancer cells. On the other hand, TNF-α-induced MMP-9 expression was dose-dependently suppressed by silibinin. To verify the regulatory mechanism of silibinin on TNF-α-induced MMP-9 expression, the gastric cancer cell lines were pretreated with silibinin prior to TNF-α. TNF-α-induced MMP-9 expression was inhibited by the MEK1/2 specific inhibitor, UO126. Finally, we investigated the effect of adenoviral constitutively active (CA)-MEK and CA-Akt on MMP-9 expression. The expression of MMP-9 was significantly increased by CA-MEK overexpression, but not by CA-Akt overexpression. Taken together, we suggest that silibinin down-regulates TNF-α-induced MMP-9 expression through inhibition of the MEK/ERK pathway in gastric cancer cells.

## Introduction

Silibinin is a major bioactive flavanone that has been isolated from milk thistle seeds, and is used for the protection against various cancers, such as skin, lung, and breast cancers [1,2,3]. Silibinin modulates the imbalance between cell survival and apoptosis through the regulation of cell cycle regulators [4]. In addition, silibinin has anti-metastasis effects by modulating specific proteins, including matrix metalloproteinases (MMPs) [4]. In a previous study we reported that silibinin down-regulates TPA-induced MMP-9 and VEGF expression through inactivation of the MAPK pathway in the MCF-7 breast cancer cell line [2]. However, the effect of silibinin has not been fully elucidated with respect to the treatment of gastric cancer. Compared with normal tissue the level of expression of tumor necrosis factor (TNF)-α is abnormally increased in pre-neoplastic lesions, such as *Helicobacter pylori*-infected gastric lesions and inflamed colonic mucosa [5,6]. TNF-α is a key cytokine involved in inflammation, immunity, and cancer development [5]. TNF-α is also able to promote angiogenesis through the induction of VEGF, VEGFR2, and bFGF [6,7]. In addition, TNF-α augments tumor remodeling with respect to tumor cell motility and tumor invasion via the induction of MMPs [8,9].

The MMPs are a major group of enzymes which can degrade nearly all components of the extracellular matrix (ECM), as well as components of basement membranes [10,11]. An elevated expression of MMPs contributes to various pathologic processes, including rheumatoid arthritis, osteoarthritis, angiogenesis, invasion, and metastasis in carcinoma [12,13,14]. Two members of the MMP family, MMP-2 and MMP-9, are highly expressed in various tumors, including breast and bladder cancer [15,16]. Serum levels of MMP-9, but not MMP-2, are significantly higher in colorectal and gastric cancer compared to controls [17]. Elevated plasma MMP-9 correlates significantly with lymph node metastasis, lymphatic invasion, venous invasion, and had poorer survival rates [18]. 

In the present study we determined the effect of silibinin on MMP-9 expression in gastric cancer cells. Our results showed that silibinin suppresses TNF-α-induced MMP-9 expression. In addition, TNF-α-induced ERK phosphorylation was significantly decreased in SNU216 gastric cancer cells by silibinin treatment. We suggest that silibinin may be an effective therapeutic drug for anti-cancer therapy by preventing cancer metastasis through the down-regulation of MMP-9 expression in gastric cancer. 

## Results and Discussion

### The basal level of MMP-9 mRNA and protein expression was increased by TNF-α in SNU216 and SNU668 gastric cancer cells in a dose-dependent fashion

**Figure 1:**
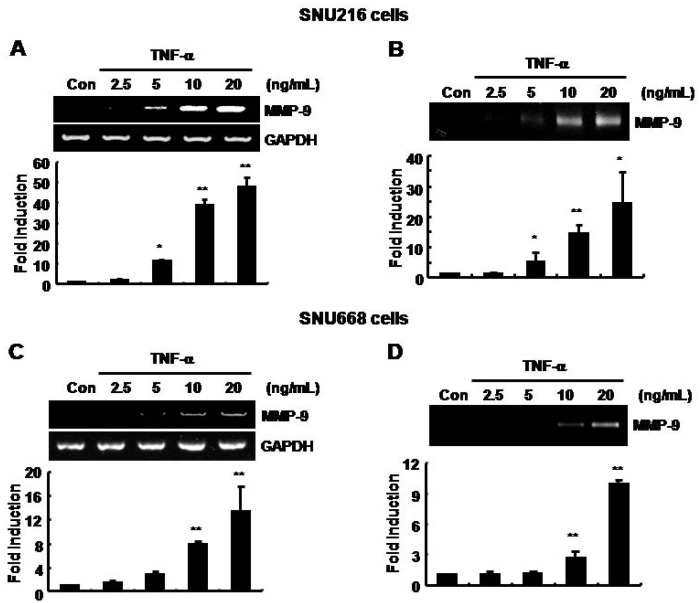
The basal levels of MMP-9 mRNA and protein expression were increased by TNF-α in a dose-dependent manner in SNU216 and SNU668 gastric cancer cells. After serum-starvation for 24 h, cells were treated with TNF-αat the concentrations indicated for 24 h in fresh serum-free media. (A, C) The level of MMP-9 mRNA expression was analyzed by RT-PCR in SNU216 and SNU668 gastric cancer cells. (B, D) The level of MMP-9 protein expression was analyzed by zymography in SNU216 and SNU668 gastric cancer cells. These results are representative of three independent experiments. The values shown are the mean ± SEM. * *P* < 0.05, ** *P* < 0.01 *vs*. control. Con; control.

To verify the effect of TNF-α on MMP-9 expression, we treated cells with TNF-α for 24 h at the indicated concentrations in SNU216 and SNU668 gastric cancer cells. The secreted MMP-9 protein and mRNA expression were measured by gelatin zymography and RT-PCR, respectively. The basal level of MMP-9 mRNA and protein expression was increased by TNF-α in a dose-dependent manner (Figure 1). In SNU216 cells, the level of MMP-9 mRNA and protein expression was increased by 47.84 ± 4.72- and 24.14 ± 10.69-fold of the control level at 20 ng/ml of TNF-α, respectively (Figure 1A and 1B). In addition, the level of MMP-9 mRNA and protein of SNU668 cells was increased by 13.37 ± 4.17- and 9.94 ± 0.28-fold of the control level following treatment with TNF-α (20 ng/ml), respectively (Figure 1C and 1D). Therefore, we demonstrated that induction of TNF-α by *H. pylori* may augment tumor metastasis through up-regulation of MMP-9 expression in gastric cancer cells.

### TNF-α-induced MMP-9 mRNA and protein expression was decreased by silibinin in SNU216 gastric cancer cells in a dose-dependent fashion

**Figure 2:**
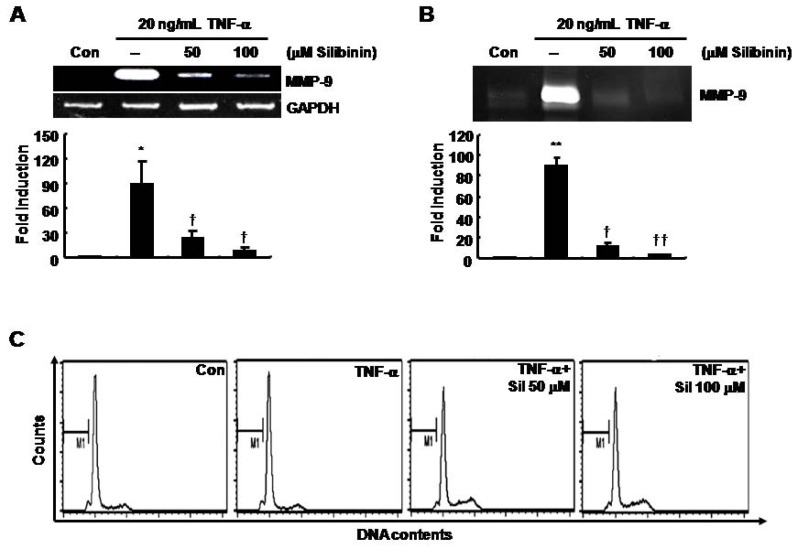
TNF-α-induced MMP-9 mRNA and protein expression was decreased by silibinin in a dose-dependent manner in SNU216 gastric cancer cells. After serum-starvation for 24 h, cells were pretreated with silibinin at the concentrations indicated for 60 min, and then treated with 20 μg/mL of TNF-α for 24 h. MMP-9 mRNA and protein expression were analyzed by RT-PCR (A) and zymography (B), respectively. (C) Under the same conditions, the cell cycle was analyzed by FACS analysis, as described in the Materials and Methods. These results are representative of three independent experiments. The values shown are the mean ± SEM. * *P* < 0.05, ** *P* < 0.01 *vs*. control, † *P* < 0.05, †† *P* < 0.01 *vs*. TNF-α-treated cells. Con; control.

To determine the effect of silibinin on TNF-α-induced MMP-9 expression, we pretreated cells with 50 or 100 μM silibinin for 60 min prior to treatment with TNF-α (20 ng/mL). TNF-α-induced MMP-9 mRNA and protein expression was decreased by silibinin in a dose-dependent fashion (Figure 2A and 2B). The level of MMP-9 mRNA was increased by 89.22±27.21-fold of the control level by treatment with 20 ng/ml of TNF-α treatment (Figure 2A). However, TNF-α-induced MMP-9 mRNA was decreased by 23.55 ± 9.42- and 8.47 ± 5.04-fold of the control level in a dose-dependent fashion following treatment with 50 and 100 μM silibinin, respectively (Figure 2A). The level of MMP-9 protein was also increased by 90.0 ± 7.68-fold of the control level with TNF-α treatment at 20 ng/mL (Figure 2B). However, TNF-α-induced MMP-9 protein expression was decreased by 10.93 ± 3.73- and 3.37 ± 0.39-fold of the control level in a dose-dependent fashion at 50 and 100 μM silibinin, respectively (Figure 2B). We next examined the effect of silibinin on the proliferation of gastric cancer cells. Under serum-starved conditions, the cell cycle was not affected by TNF-α and/or silibinin treatment (Figure 2C). Therefore, we demonstrated that silibinin may be a therapeutic drug for metastasis of gastric cancer through down-regulation of MMP-9 expression. 

### TNF-α-induced MMP-9 expression was decreased by UO126 and LY294002 in SNU216 gastric cancer cells

**Figure 3:**
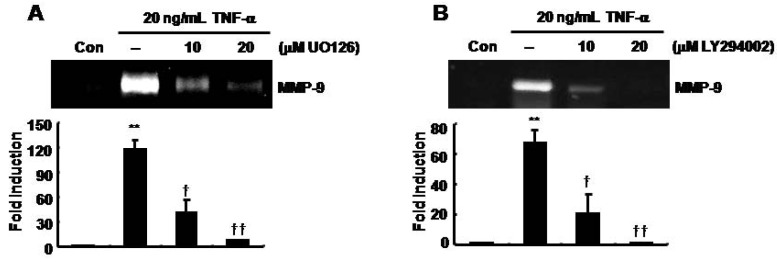
TNF-α-induced MMP-9 expression was decreased by UO126 and LY294002 in SNU216 gastric cancer cells. After serum-starvation for 24 h, cells were pretreated with UO126 (A) and LY294002 (B) at the indicated concentrations for 30 min and then treated with 20 μg/mL TNF-α for 24 h. MMP-9 gelatinase activity was analyzed in culture media by zymography. These results are representative of three independent experiments. The values shown are the mean ± SEM. ** *P* < 0.01 *vs*. control, † *P* < 0.05, †† *P* < 0.01 *vs*. TNF-α-treated cells. Con; control.

The effect of selective MEK1/2 and PI-3 kinase inhibitors on TNF-α-induced MMP-9 expression was determined. We pretreated SNU216 cells with UO126 (a MEK1/2 inhibitor) and LY294002 (a PI-3 kinase inhibitor) at the indicated concentrations prior to treatment with 20 ng/ml of TNF-α for 30 min, and then continued the incubation for an additional 24 h. TNF-α-induced MMP-9 expression was inhibited by UO126 in a dose-dependent manner (Figure 3A). The level of MMP-9 expression was increased 117.93 ± 11.17-fold of the control level at 20 ng/ml of TNF-α (Figure 3A). In contrast, TNF-α-induced MMP-9 expression was decreased by 40.45 ± 15.43- and 7.01 ± 0.18-fold of the control level in a dose-dependent fashion following treatment with 10 and 20 μM UO126, respectively (Figure 3A). 

In addition, TNF-α-induced MMP-9 expression was inhibited by LY294002 (Figure 3B). TNF-α-induced MMP-9 expression was inhibited 20.91 ± 12.67- and 0.96 ± 0.03-fold of the control level following treatment with 10 and 20 μM LY294002, respectively (Figure 3B). Therefore, we demonstrated that silibinin suppressed TNF-α-induced MMP-9 expression through inhibition of the MAPK and PI-3 kinase pathway in gastric cancer cells.

### MMP-9 expression was up-regulated by adenoviral constitutively active-MEK overexpression (CA-MEK), but not by CA-Akt, in SNU216 gastric cancer cells

**Figure 4:**
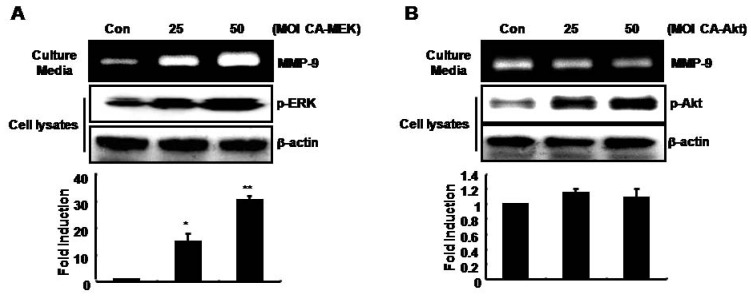
TPA-induced MMP-9 expression is increased by Ad-CA-MEK, but not by Ad-CA-Akt, in SNU216 gastric cancer cells. After Ad-CA-MEK (A) and CA-Akt (B) transfection for 24 h, cells were incubated in serum-free media for 24 h and then fresh serum-free media was added for 24 h. MMP-9 protein expression was analyzed in culture media by zymography. Using the cell lysates, the phosphorylation of ERK (A) and Akt (B) was analyzed by Western blotting. These results are representative of three independent experiments. The values shown are the mean ± SEM. * *P* < 0.05, ** *P* < 0.01 *vs*. control. Con; control.

Finally, we investigated the regulatory mechanism of MMP-9 expression by the MAPK and PI-3 kinase pathways. Then, we transfected cells with CA-MEK and CA-Akt for 24 h, followed by incubation for an additional 24 h in fresh serum-free media. After 24 h, we harvested the culture media and cell lysates. As shown in Figure 4A, MMP-9 expression was increased by CA-MEK in a dose-dependent manner. In cell lysates, the phosphorylation of ERK, a downstream target of MEK, was also increased by CA-MEK overexpression (Figure 4A). On the other hand, MMP-9 expression was not affected by CA-Akt overexpression (Figure 4B). Therefore, we demonstrated that silibinin prevented TNF-α-induced MMP-9 expression via suppression of the MAPK pathway in gastric cancer cells.

Silibinin is a major bioactive flavanone that has been isolated from milk thistle seeds, and has been used as a traditional medicine [1]. Silibinin also suppresses the invasion and motility of cancer cells by down-regulating MMP-2 and up-regulating TIMP-2 expression [19,20]. Silibinin exerts an inhibitory effect on the expression of TNF-α and other pro-inflammatory cytokines, including IFN-γ, IL-4, and IL-2, as well as TNF-α signaling [21,22]. Silibinin also inhibits the invasion, motility and migration of prostate cancer cell lines via down-regulation of vimentin and MMP-2 [23]. In addition, Lin *et al*. reported that silibinin also inhibits cell growth and protein translation through suppression of mTOR activity in MCF7 breast cancer cells [24].

*H. pylori* has been shown to be a potent carcinogen in human gastric cancer by the International Agency for Research on Cancer (IARC) [25]. It has been reported that infection with *H. pylori* augments the inflammation associated with the induction of TNF-α and other inflammatory cytokines, such as IL-1β and IL-10, in the stomach [26]. In addition, TNF-α increases the risk for chronic atrophic gastritis and gastric carcinoma [27].

TNF-α is a key pro-inflammatory cytokine and is secreted by activated macrophages and monocytes [28]. TNF-α has been implicated in various human diseases, such as rheumatoid arthritis, septic shock, and tumorigenesis [29,30]. TNF-α is elevated in various human cancers and has a positive correlation with tumor grade and poor prognosis [31,32]. Therefore, we investigated the role of silibinin as a therapeutic drug for inhibition of TNF-α-induced MMP-9 expression, which is a hallmark of cancer metastasis and invasion in gastric cancer cells. 

MMP-9 is known to specifically cleave type IV collagen, which is the major component of basement membranes [33]. The MMP-9 promoter has several transcription factor-binding motifs, including the AP-1, Sp-1, and NF-κB binding sites [33]. These sites are required for stimulation in response to TNF-α [34,35]. In a previous study we also reported that TNF-α-induced MMP-9, and cell invasion is significantly decreased by berberine through inhibition of AP-1 activity in MDA-MB-231 human breast cancer cells [34]. TNF-α-induced MMP-9 expression is also decreased via inhibition of the MAPK pathway, such as the ERK and p38 pathways in human urinary bladder cancer cells [36]. In agreement with these reports, our results showed that TNF-α-induced ERK phosphorylation and MMP-9 expression is decreased by silibinin treatment. The MEK1/2 inhibitor, UO126, also completely blocked TNF-α-induced MMP-9 expression in SNU216 gastric cancer cells. On the other hand, the basal level of MMP-9 expression was significantly increased by CA-MEK overexpression. However, the phosphorylation of p38 had no affect by TNF-α. Therefore, we suggest that TNF-α-induced MMP-9 expression is mediated through a MEK/ERK-dependent pathway. Then, silibinin suppresses TNF-α-induced MMP-9 expression through inhibition of the MEK/ERK pathway.

In addition, heregulin-β-mediated activation of MMP-9 is blocked by PKC (RO318220), p38 (SB203580), and MEK-1 inhibitors (PD98059), but not a PI-3 kinase inhibitor (wortmannin) [37]. However, Hwang *et al*. recently reported that TNF-α-induced MMP-9 up-regulation and cell migration is associated with the PI3K/Akt pathway in the JB6 mouse epidermal cell line [38]. We also observed that TNF-α-induced MMP-9 expression is inhibited by another PI-3 kinase inhibitor, LY294002, in a dose-dependent manner. To confirm these results, we determined the effect of CA-Akt on the basal level of MMP-9 expression. As shown in Figure 4B, CA-Akt overexpression had no affected on the level of MMP-9 expression in SNU216 and SNU668 (data not shown) gastric cancer cells. Therefore, we suggest that Akt activity does not directly affect TNF-α-induced MMP-9 expression in gastric cancer cells.

## Conclusion

In conclusion, we determined the effect of silibinin on TNF-α-induced MMP-9 expression in gastric cancer cells. We suggest that silibinin inhibits TNF-α-induced MMP-9 expression through inactivation of the MEK/ERK pathway. Silibinin may be an effective additive to anti-metastatic therapy by preventing cancer metastasis through the down-regulation of MMP-9 in gastric cancer. 

## Experimental

### Reagents and cell cultures

Cell culture media, antibiotics, and 10% Zymogram gel were purchased from Life Technologies (Rockville, MD, USA). Rabbit polyclonal anti-phospho-ERK and anti-Akt antibodies were purchased from Epitomics (Burlingame, CA, USA). TNF-α was purchased from Biosources (Minneapolis, MN, USA). Silibinin [2,3-dihydro-3-(4-hydroxy-3-methoxyphenyl)-2-(hydroxymethyl)-6-(3,5,7-trihydroxy-4-oxobenzopyran-2-yl)benzodioxin] was purchased from Sigma (St. Louis, MO, USA). The secondary peroxidase-conjugated antibodies and ECL reagents were from Amersham (Buckinghamshire, UK). 

The human gastric cancer cell lines, SNU216 and SNU668, were cultured in RPMI1640 supplemented with 10% fetal bovine serum (FBS), 2 mM glutamine, 100 IU/ml penicillin, and 100 μg/ml streptomycin. Each cell was maintained in culture medium supplemented without FBS for 24 h.

*Cell proliferation* assay: Total cell numbers were evaluated by a Quick Cell Proliferation Assay Kit II (BioVision, Mountain View, CA, USA), according to the manufacturer’s protocol. Briefly, cells (5 × 10^4^/well) were cultured in a 96-well plate in 100 μL/well of culture media in the presence or absence of various quantities of silibinin. After incubating the cells for 24 h, 10 μL of WST reagent was added to each well. Viable cells were quantified photometrically at 480 nm.

*Chemical inhibitor treatment*: Cells were maintained in culture medium without FBS for 24 h, and then the culture media were replaced with fresh media without FBS and the cells were further incubated with various inhibitors, including 10 μM UO126 and LY294002 for 24 h. In experiments involving these inhibitors, cells were pretreated for 30 min prior to treatment with TNF-α (20 ng/mL). 

*RT-PCR*: Total RNA was extracted from SNU216 and SNU668 human gastric cancer cells using TRIzol (Invitrogen, Carlsbad, CA, USA) according to the manufacturer's protocol. Equal amounts of RNA (1 μg) were reverse-transcribed using a first-strand cDNA synthesis kit (MBI Fermentas, Vilnius, Lithuania). Semi-quantitative PCR was performed using the following specific primers: human MMP-9 forward, CCC GGA CCA AGG ATA CAG; and reverse, GGC TTT CTC TCG GTA CTG; and as an internal control, GAPDH forward, ATT GTT G CC ATC AAT GAC CC; and reverse, AGT AGA GGC AGG GAT GAT GT. The PCR conditions were 1 cycle of initial denaturation for 5 min at 94 °C, 21 cycles (GAPDH) or 30 cycles (MMP-9) of amplification for 1 min at 94 °C, 1 min at 60 °C, and 1 min at 72 °C, and 1 cycle of final extension for 10 min at 72 °C. The reaction products were electrophoresed in 2% agarose gels and visualized with ethidium bromide (EtBr). Signal densities were quantified using a densitometric program (Bio 1D; Vilber Lourmat, Marne La Vallec, France). 

*Zymography*: Zymography was performed in 10% polyacrylamide gels that had been cast in the presence of gelatin, as described previously [39]. Briefly, samples (culture media) were resuspended in loading buffer and run on a 10% SDS-PAGE gel containing 0.5 mg/mL gelatin without prior denaturation. After electrophoresis, gels were washed to remove SDS and incubated for 30 min at room temperature (RT) in a renaturing buffer (50 mM Tris, 5 mM CaCl_2_, 0.02% NaN_3_, and 1% Triton X-100). The gels were then incubated for 48 h at 37 °C in a developing buffer (50 mM Tris-HCl [pH 7.8] 5 mM CaCl_2_, 0.15 M NaCl, and 1% Triton X-100). The gels were subsequently stained with coomassie brilliant blue G-250 and destained in 30% methanol, and 10% acetic acid to detect gelatinase secretion. Signal densities were quantified using a densitometric program (Bio 1D; Vilber Lourmat, Marne La Vallec, France). 

*Western blotting*: Cell lysates were used in immunoblot analysis for ERK1/2, Akt, and β-actin. Proteins were boiled for 5 min in Laemmli sample buffer and electrophoresed in 10% SDS-PAGE gels. Proteins were transferred to PVDF membranes and the membranes were then blocked with 10% skim milk in TBS with 0.01% Tween-20 for 15 min. The blots were incubated with anti-phospho-ERK and Akt antibodies (1/1,000 dilution) in 1% TBS/T buffer (0.01% Tween 20 in TBS) at 4°C overnight. Blots were washed 3 times in TBS with 0.01% Tween 20 and subsequently incubated in anti-rabbit peroxidase-conjugated antibody (1/2,000 dilution) in TBS/T buffer. After 1 h incubation at RT, the blots were washed three times and ECL reagents (Amersham Bioscience) were used for development. Signal densities were quantified using a densitometric program (Bio 1D; Vilber Lourmat, Marne La Vallec, France). 

*Flow cytometry analysis (FACS)*: Cells were trypsinized and harvested by centrifugation at 1,500 rpm for 5 min. The cell pellets were then resuspended in 1mL PBS and fixed in 70% ethanol for 20 min at room temperature (RT). Fixed cells were centrifuged, and washed twice in PBS to wash out any apoptotic cells. The cells were resuspended in 1 mL of PBS with 100 µg/mL of DNase-free RNase A (Biopure, Canada), then incubated for 30 min in a 37 °C water bath. The cells were collected by centrifugation at 1,500 rpm, the cell pellets were washed twice with PBS, resuspended in PBS containing 50 µg/mL of propidium iodide (Sigma), then analyzed using the FACS-vantage (Becton–Dickinson, San Diego, CA, USA).

*Adenovirus transfer*: Adenoviral human constitutively active (CA)-MEK and CA-Akt cDNA were generous gifts from Dr. Hyunil Ha (Seoul National University, Korea). For infection, recombinant adenovirus was diluted in RPMI-1640 containing 10% FBS, and added to the cells at 37 °C for 24 h. After Ad-CA-MEK and CA-Akt transfection, the media were replaced with serum-free media for 24 h. The expression of construct was confirmed by Western blotting against phospho-ERK and phospho-Akt.

*Statistical analysis:* Statistical significance was determined using Student’s t-test. The results are presented as the mean ± SEM. All *p*-values were two-tailed and the significance was accepted at a *p* < 0.05.
